# Prevalence, risk factors and clinical implications of malnutrition in French Comprehensive Cancer Centres

**DOI:** 10.1038/sj.bjc.6605578

**Published:** 2010-02-16

**Authors:** M Pressoir, S Desné, D Berchery, G Rossignol, B Poiree, M Meslier, S Traversier, M Vittot, M Simon, J P Gekiere, J Meuric, F Serot, M N Falewee, I Rodrigues, P Senesse, M P Vasson, F Chelle, B Maget, S Antoun, P Bachmann

**Affiliations:** 1 Department of Anesthesiology, Institut Claudius Regaud, 20–24 rue du Pont Saint-Pierre, Toulouse 31052, France; 2 Department of Medico-economics, Institut Claudius Regaud, 20–24 rue du Pont Saint-Pierre, Toulouse 31052, France; 3 CLCC Interclan, Unit of Nutrition, Centre Léon Bérard, 28 rue Laennec, Lyon 69008, France

**Keywords:** malnutrition, weight loss, BMI, risk factor, obesity

## Abstract

**Background:**

This epidemiological observational study aimed at determining the prevalence of malnutrition in non-selected adults with cancer, to identify risk factors of malnutrition and correlate the results with length of stay and 2-month mortality.

**Methods:**

This prospective multicentre 1-day study conducted in 17 French Comprehensive Cancer Centres included 1545 patients. Body mass index (BMI), weight loss (WL) in the past 6 months and age were routinely recorded according to the French national recommendations for hospitalised patients; malnutrition was rated as absent, moderate or severe according to the level of WL and BMI. Age, sex, tumour site, type of hospitalisation and treatment, disease stage, World Health Organisation performance status (PS) and antibiotic therapy were the potential malnutrition risk factors tested. Follow-up at 2 months allowed to determine the correlation with length of stay and mortality.

**Results:**

Malnutrition was reported in 30.9% of patients, and was rated as severe in 12.2%. In multivariate analysis, only pre-existing obesity (BMI⩾30), PS ⩾2 and head-and-neck or upper digestive cancers were associated with increased risk of malnutrition. Antibiotics use was significantly higher in malnourished patients (35.5 *vs* 22.8% *P*<0.001). Severe malnutrition was independently associated with mortality. The median length of stay was 19.3±19.4 days for malnourished patients *vs* 13.3±19.4 days for others (*P*<0.0001).

**Conclusion:**

In French Comprehensive Cancer Centres, one out of three cancer patients are malnourished and this was associated with a longer length of stay. Pre-existing obesity could be identified as a new risk factor for malnutrition in our cancer patient population perhaps because of a misidentification or a delay in nutrition support in this category of patients.

In cancer patients, malnutrition and weight loss (WL) have been identified as being associated with worse outcome, impaired quality of life and performance status (PS). In the often-cited study by Dewys *et al* (1980), WL before treatment is reported in 54% of 3047 patients enrolled in 12 chemotherapy protocols and is linked to the World Health Organisation (WHO) PS and outcome. Many reviews have highlighted this high prevalence of malnutrition in cancer patients, its potential for adverse effects on outcome and its economic consequences (Nitenberg and Raynard, 2000; Norman *et al*, 2008). On the other hand, published data of malnutrition prevalence in populations of cancer patients are often given as a function of localisation (Dewys *et al*, 1980; Bozzetti, 2009), tumour stage (Andreyev *et al*, 1998; Segura *et al*, 2005) or treatment (Dewys *et al*, 1980). Other prevalence data are available from recent surveys of hospitalised patients, but cancer patients generally represent only a limited proportion of the study population, usually one in four (Correia and Waitzberg, 2003; Pirlich *et al*, 2006) or even less (Kruizenga *et al*, 2003). Very few surveys have explored large non-selected populations of cancer patients treated at specialised centres in Europe (Nourissat *et al*, 2007).

The objective of this epidemiological observational multicentre ‘one-day’ study in non-selected hospitalised adults with cancer was to determine the prevalence of malnutrition during hospitalisation in cancer centres and to identify potential risk factors for malnutrition (such as age, sex, tumour site, type of hospitalisation and treatment, disease, PS and antibiotic therapy). Patient follow-up at 2 months was used to determine the association between malnutrition, length of hospital stay (LOS) and mortality.

## Materials and methods

This prospective epidemiological observational multicentre study was conducted in voluntary cancer centres in France. Between October 2007 and January 2008, each cancer centre chose 1 day to conduct the study, except Mondays and Fridays (usually associated with a higher number of hospital admissions and discharges) and weekends. All adult hospitalised patients from every unit or ward were included on the same day. Patients admitted for 1-day hospitalisation (outpatient clinic) were also eligible if possible. The only exclusion criteria were age below 18 years, absence of malignant diagnosis at the end of stay and patients in agony. Owing to a lack of staff to evaluate all patients admitted at a given centre, it was possible to conduct the study in only a limited number of wards, but an exhaustive investigation of all patients was performed. The study was observational and required no particular intervention; therefore, it was not subjected to ethics committee agreement (Claudot *et al*, 2008). The database was registered with the French national authorities (CNIL, Commission Nationale de l’Informatique et des Libertés). Computerised data were processed anonymously. Information was given to all patients on the day of the study.

Malnutrition was defined following the recommendation of the French health authority (Haute Autorité de Santé) (www.has-sante.fr/portail/upload/docs/application/pdf/denutrition _personne_agee_2007_recommandations.pdf) (www.has-sante.fr/portail/upload/docs/application/pdf/ denutrition_rap_2006_09_25_14_20_46_269.pdf) and by the Nutricode labelled by the French society of parenteral and enteral nutrition 2006 (http://www.nutricode.fr/) using age (in years), BMI (in kg m^−2^) and WL (in percentage over the previous 6 months). Malnutrition was rated as absent, moderate or severe (Table 1). Patients’ weight (*W*) and height (*H*) were recorded during the hospital stay, as usually recommended in France for hospitalised patients. If not applicable, inability to measure weight or height was reported. When it was not possible to measure the patient's height, knee height measurement was performed and patient stature was calculated using the following formulas (Chumlea *et al*, 1985):

- For women: *H*=84.88−(0.24 × age in years)+(1.83 × knee height in centimetres) and
- for men: *H*=64.19−(0.04 × age in years)+(2.02 × knee height in centimetres)

Patients were asked their weight 6 months before the study. When they did not remember or were uncertain about it, information was retrieved from patient records; when available, the values were used for calculation of WL in percentage (%) using the following ratio: ((*W* 6 months earlier−current *W*)/100 × *W* 6 months earlier). Body mass index (BMI) was also calculated, such as BMI=*W*/*H*^2^ in kg m^−2^.

Other data collected on the day of the study were the following: patient's birth date and gender, type of hospitalisation (conventional or outpatient), site of primary tumour, presence of distant metastasis (yes or no), treatment received during the stay or in relation to current hospitalisation (surgery, radiotherapy, chemotherapy), prescription of antibiotics during the stay until the day of study (with the exception of antibiotic prophylaxis for surgery) and type of nutritional support until the study day (dietetic counselling, enteral or parenteral nutrition). Treatment was defined as active when patients received active cancer treatment with intention to cure or to obtain a remission (radiotherapy or chemotherapy within 1 month, and surgery during the stay); many patients could be classified as receiving active treatment even if they had metastatic disease. Hospitalisation for a complication of an active treatment was also classified as active. Treatment was considered palliative when patients received treatment (sometimes with anti-neoplastic and/or only supportive therapies) to relieve symptoms in the course of a progressive disease. Finally, disease was considered terminal when patients were likely to die within 1 month. Patients were considered ‘under evaluation’ when the decision for anti-neoplastic treatment was not actually made and the patient was still undergoing diagnostic testing. Performance status was determined on the day of the study using the definition proposed by WHO. For patients recovering from recent surgery, the value of PS at admission was possibly considered.

Two months after the study day, we determined the LOS corresponding to the duration of stay from admission to discharge, or to the day of the study plus 60 days if the patient was still in hospital at that time. At this time, the patient status (alive, dead or unknown) was also determined. Patients admitted for 1-day hospitalisation were excluded from LOS analysis.

When BMI was not indicative of the presence of malnutrition (>18.5 or >21 before or after 70 years of age, respectively) and WL could not be determined (absence of weight data 6 months earlier), we considered that malnutrition could not be eliminated and patients were not analysed for the association with risk factors or outcomes.

For descriptive analyses, qualitative data were summarised as frequencies, and results for continuous data were expressed as means and s.d. Association between malnutrition and clinical status was assessed using the *χ*^2^ test or Fisher's exact test and analyses of variance for categorical and continuous measurements, respectively. A *P*-value of <0.05 was considered statistically significant. Backward stepwise logistic regression analysis was performed on variables associated with *P*<0.20. Results were considered statistically significant when *P*-values were <0.05. The same analysis was repeated to identify risk factors for mortality, and a logistic regression was used to determine whether malnutrition was an independent factor. Statistical analyses were performed using STATA software (release 8.0, Stata Corporation, College Station, TX, USA).

## Results

A total of 1545 patients, 885 women (57.2%) and 660 men (42.8%), were included. The median age was 59.3±13.8 years, with 361 (23.4%) patients older than 70 years. The most frequent tumour sites were the breast (24%), and the head and neck (12%); 825 (53%) patients had localised cancer, whereas 720 (47%) had metastatic disease. Despite this metastatic status, most patients (80%) experienced active treatment. Patient and treatment characteristics are presented in Tables 2A and 2B.

The overall prevalence of malnutrition was 30.9%, with 18.6 and 12.2% cases of moderate or severe malnutrition, respectively. In addition, 60.4% of patients reported a WL in the previous 6 months. Nutritional status could not be determined in 181 (12%) patients, principally because there was no information regarding their weight 6 months before the study. As mentioned in the ‘Materials and methods’ section, a normal BMI on the study day was not considered sufficient to confirm the absence of malnutrition because of the relatively low sensitivity of this indicator, which identified only 12.4% of our 30.9% patients with malnutrition. The nutritional status of patients is described in detail in Table 2A. Briefly, 62% of malnourished patients received nutritional support (*vs* 31.7% in the absence of malnutrition; *P*<0.001); this support included dietetic counselling alone (49.2%) or the use of oral supplementation or artificial nutrition (12.8%).

The results of univariate analysis presented in Table 3 indicate that male gender, presence of metastases, inpatient hospitalisation, palliative care and radiotherapy are associated with the presence of malnutrition. Obese patients (BMI ⩾30, 6 months earlier) were more prone to malnutrition (38.8 *vs* 28.5% *P*<0.01); in these patients, only the risk of severe malnutrition seemed significant (OR=2.26; 95% CI (1.5–3.4); *P*<0.0001). The prevalence of malnutrition was moderately associated with the WHO PS, with a major increase in patients with PS⩾ 2. Finally, antibiotics intake was significantly increased in malnourished patients (35.5 *vs* 22.8% *P*<0.001).

In multivariate analysis (Table 4), only obesity at 6 months before the study, poor functional status (PS⩾2) and head-and-neck or upper digestive cancers were independently associated with malnutrition.

Follow-up data at 2 months were available for 1081 patients. Mortality (18.4%) was significantly higher in malnourished patients than in the other group (26.7 *vs* 11.8% *P*<0.0001; OR 2.7 (1.9–3.9)), especially in patients diagnosed with severe malnutrition (37.1% OR 4.4 (2.8–6.9)) compared with those with mild symptoms (20.2% OR 1.9 (1.2–2.9)). Mortality was also higher in patients for whom no weight or height information was available (25.7 *vs* 17.6% *P*=0.045). A multivariate analysis taking into account major confounding factors such as age, gender, type of stay, type of cancer, treatment, presence or absence of metastases, antibiotics intake and PS showed that only severe malnutrition was independently associated with mortality (Table 5).

The LOS was available for 879 inpatients. Malnutrition, either moderate or severe, was significantly associated with prolonged LOS. The median LOS was 19.3±19.4 days for malnourished patients *vs* 13.3±19.4 days for others (*P*<0.0001). Patients for whom no information on malnutrition status was available had an LOS of 19.5±20.8 days, which was similar to results obtained in malnourished patients. Patient nutritional status did not remain significant when compared with other confounding factors possibly associated with prolonged LOS. Only PS, head-and-neck cancers, haematological malignancies and terminal stage remained significantly associated with prolonged LOS (results not shown).

## Discussion

In this prospective observational study, the prevalence of malnutrition, defined as a function of two anthropometric indicators, BMI and WL was 30.9%. This result applied to patients from comprehensive cancer centres that are considered as expert centres and may thus treat patients with more advanced cancers. The most recent data in Europe are those of the German hospital malnutrition study of 475 cancer patients (of 1886 hospitalised patients) published in 2006 (Pirlich *et al*, 2006). Using subjective global assessment, the German investigators have rated patients as malnourished (SGA B) or severely malnourished (SGA C), with malnutrition rates of 37.6%. The median age was 63±14 years and 56% of patients were men, which is higher than that in this study. A Dutch study published in 2001 included 1186 cancer patients (in 7606 patients, of whom 81% were hospitalised) (Kruizenga *et al*, 2003). The prevalence of malnutrition, defined as a >10% WL in the previous 6 months, was 21%. A French study conducted in 2006 in 477 cancer patients has reported WLs>10% in 6 months (or >5% in 1 month) in 22.4% of patients (Nourissat *et al*, 2007), whereas rates of 39.7% have been reported by Bozzetti (2009) in an Italian population of 1000 patients with selected cancers (digestive, lung or head-and-neck cancers). The higher risk of malnutrition associated with tumours of the upper digestive tract or head-and-neck cancers described in this study is in agreement with that of most previous studies (Nitenberg and Raynard, 2000; Kruizenga *et al*, 2003; Nourissat *et al*, 2007, Bozzetti, 2009). Prevalence is generally lower in patients with breast cancer. This population represented 24% of our study sample; 18.3% were found malnourished, or even severely malnourished, as 12.3% had ⩾10% WL, which is more than twice the rate reported by Dewys *et al* (1980) for patients investigated at the beginning of chemotherapy. However, this high prevalence could be due to the fact that 44% of our breast cancer patients had metastatic disease. In this study, as in most papers describing the epidemiology of malnutrition in large cancer patient populations, a limitation could arise from the validity of the parameters used to define malnutrition but, indeed, WL and low BMI are commonly used and associated with outcome.

Low BMI was reported in 12.4% of our patients; however, only 7.3% of malnourished patients were diagnosed with this parameter and, despite a significant WL (<10%), many patients could not be classified as malnourished. Low BMI is thus not significantly correlated with malnutrition. This is in agreement with several other authors who have reported that only 10% of malnourished patients are detected when using the BMI criterion, *vs* 30–40% when using the WL criterion (Kruizenga *et al*, 2003; Nourissat *et al*, 2007). However, Kruizenga *et al* (2003) have suggested that a low BMI is often associated with malnutrition (OR=6.01; 95% CI (4.92–7.33)), even if the correlation between WL and BMI is poor and the discriminative power of the test is low. This criterion thus remains relevant for several reasons: calculating BMI can be used to (1) detect malnourished patients in the absence of WL (one in four of the 30.8% malnourished patients identified in this study); (2) select specific populations at risk of increased mortality, such as elderly patients with low BMI (Landi *et al*, 2000); and (3) identify obese patients shown to be potentially at higher risk of malnutrition.

Malnutrition in this study has several negative consequences. First, it is associated with functional impairment, in agreement with the literature (Dewys *et al*, 1980; Bozzetti, 2009). It is also linked to other indicators associated with increased cost burden on the health-care system. In univariate analysis, the need for antibiotics was 1.87 higher in malnourished patients (*P*<0.001), but this criterion did not remain significant in multivariate analysis. Schneider *et al* (2004), who have examined the correlation between nutritional status (evaluated using the nutritional risk index NRI) and nosocomial infections, have shown that the risk of infection is increased in patients with moderate malnutrition (OR 1.46; 95% CI (1.2–2.1)), and especially in those suffering from severe malnutrition (OR 4.98; 95% CI (8.8–12.6)).

Malnutrition is also frequently associated with longer hospital stays, which are indicative of higher costs (Norman *et al*, 2008). In this study, the length of stay was found to be increased by 45% in malnourished patients. This result is close to the 42% reported by Pirlich *et al* (2006) in the German study, which included 25% of patients with cancer, whereas other authors have reported even higher increases (60%) in populations including 28% of cancer patients (Correia and Waitzberg, 2003). Contrary to PS, length of stay did not remain significantly correlated with malnutrition after adjustment for potentially confounding factors. However, it is generally admitted that nutritional support can reduce the LOS and is consequently cost-effective for malnourished patients (Tucker and Miguel, 1996; Johansen *et al*, 2004; Kruizenga *et al*, 2005).

The correlation between mortality and malnutrition is considered to be very high in cancer patients (Norman *et al*, 2008). Dewys *et al* (1980) have evidenced an impact of malnutrition on outcome in patients with only moderately impaired PS or with limited tumour burden. Results of this study confirmed the prognostic impact of the common factors independently associated with mortality: PS, age >70 years, metastatic disease, some tumour sites (blood, gynaecologic organs, lung, etc.) or the reason for hospital admission (palliative care or evaluation). Severe (but not moderate) malnutrition was found to be significantly correlated with mortality (OR 2.47; 95% CI (1.4–4.36); *P*=0.002). Finally, patients for whom no weight and height information was available were found to have higher mortality in univariate analysis, but in these patients, PS is also higher (results not shown). Similar information was obtained by Izawa *et al* (2007) in frail elderly patients.

Indeed, in the present population, the major observation was that obesity (BMI ⩾30) 6 months before the study was associated with an increased risk of malnutrition (OR 1.55; 95% CI (1.06–2.27); *P*=0.024). Obesity is a well-known risk factor in many of the most prevalent tumours (Calle *et al*, 2003). Obesity has also been considered as a factor of poor prognosis in many studies (Dignam *et al*, 2006; Cleveland *et al*, 2007; Majed *et al*, 2008; Li *et al*, 2009). Particularly in patients with breast cancer, obesity may be a reason for under-treatment because treatment doses are not always adjusted to actual weight (Griggs *et al*, 2005). However, insufficient treatment is certainly not the only factor linking bad prognosis to overweight and obesity during cancer treatment, and the relationship between obesity, adipose tissue function, inflammation, insulin resistance and tumour growth is a major field of research (McTiernan, 2005; van Kruijsdijk *et al*, 2009). Although it is recognised that weight stabilisation during chemotherapy is associated with improvement in survival (Andreyev *et al*, 1998; Ross *et al*, 2004), it is unlikely that patients included in our study population were asked to lose weight and that they have voluntarily done so. The recent guidelines recommend to prevent therapy-associated WL during therapy (Arends *et al*, 2006). More probably, patients with high BMI were those more frequently exposed to significant WL and malnutrition because patients and caregivers paid less attention to this loss in case of obesity. Recently, Prado *et al* (2008) have suggested that 15% of obese patients have sarcopaenia, a complication associated with poorer functional status and independently predictive of mortality (HR 4.2; 95% CI (2.4–4.7)). In this study, the fact that malnutrition (mainly estimated from WL) was associated with a poor functional status is probably related to the occurrence of sarcopaenia. Hence, obesity, which is a growing concern in the Western world and a major risk factor for life-threatening diseases, is also perhaps associated with a higher risk of malnutrition in cancer patients. With the growing population of overweight and obese patients, it will become a major challenge in the next decade to better diagnose malnutrition, to develop new techniques to adapt treatment to adequate body composition parameters (Prado *et al*, 2007) and eventually to promote voluntary WL in severely obese patients, without loss of lean body mass and impairment of the functional status.

## Conclusions

The data reported in this study confirm the high prevalence of malnutrition in cancer patients (one out of three patients). This morbidity related to disease or to treatment is associated with an impaired functional status, more frequent use of antibiotics and higher mortality. The economic consequences for hospitals are substantial; the LOS is 45% longer for malnourished patients than for others, most likely owing to poorer PS (high PS score). This is also the first report of obesity as a possible risk factor for malnutrition in a large non-selected population of cancer patients. This information should be confirmed in future studies and the mechanisms involved should be further explored, especially because caregivers often fear that obesity may be associated with underlying nutritional deficiency.

## Figures and Tables

**Table 1:** Malnutrition, definition

**Moderate malnutrition**	**Severe malnutrition**
Age ⩽70 years	Age ⩽70 years
Weight loss over the past 6 months ⩾10% or BMI <18.5	Weight loss over the past 6 months ⩾15% or BMI <16
Age >70 years	Age >70 years
Weight loss over the past 6 months ⩾10% or BMI <21	Weight loss over the past 6 months ⩾15% or BMI <18

Abbreviation: BMI=body mass index.

**Table 2A:** Patient characteristics and type of disease

**Patient characteristics**	**Total**	**Breast cancer**	**Head and neck cancer**	**Colorectal cancer**	**Haematological malignancy** a	**Gynaecological cancer** b	**Upper digestive cancer** c	**Lung cancer**	**Others** d
(Number) %	(1545) 100%	(375) 24.3%	(179) 11.6%	(156) 10.1%	(156) 10.1%	(137) 8.9%	(103) 6.7%	(90) 5.8%	(349) 22.6%
Age (years)	59.3±13.8	58.2±12.7	59.4±9.8	64.6±12.5	57.3±16.8	59±12.7	62.6±11	60.2±11.2	58±16.6
>70 years	23%	19.7%	13.4%	34%	24.3%	19.7)	26.2%	18.9%	13.4%
M/F ratio	0.746	0.011	3.48	0.95	1.48	0	2.43	1.43	0.75
Metastases	46.6%	44.3%	24%	63.5%	16%	60.6%	50.5%	81%	51.3%
Outpatient/in-patient	15.6/84.4%	24.9/75.1%	6.2/93.8%	28.2/71.8%	9.2/90.8%	12.7 /87.3%	14.8/85.2%	9.6/90.4%	10.1/89.9%
									
WHO PS 0–1	49.8%	64.2%	47%	51.3%	40.7%	46.4%	45%	26.4%	48.4%
WHO PS 2–4	50.2%	35.8%	53%	48.7%	59.3%	53.6%	55%	73.6%	51.6%
									
*6 Months WL*									
No	39.6%	53.7%	25.8%	31.6%	43.7%	39.3%	21%	34.6%	40.3%
0% > WL <5%	19.5%	22.1%	17.6%	20.9%	13.4%	15.6%	15.8%	16%	23%
5% ⩾ WL <10%	17.4%	11.9%	19.5%	24.5%	17.9%	17.2%	19%	19.8%	18%
10% ⩾ WL <15%	12.6%	6.9%	17.6%	14.4%	17%	13.1%	22.1%	11.1%	11.2%
>15%	10.9%	5.4%	19.5%	8.6%	8%	14.8%	22.1%	18.5%	7.5%
									
*Current BMI*	24.1±4.7	24.7±4.7	22.7±4.5	24.1±4.1	24.9±4.8	24.5±5	22.8±4.3	24.9±4.8	24.2±4.8
<18.5 + ⩽70 years	8.4%	5.2%	15.1%	6%	5.6%	8%	12%	15.3%	15.1%
<21 + >70 years	4%	3.6%	2.3%	7.4%	2.1%	1.6%	5%	4.7%	2.3%
%BMI ⩾ 30	11.1%	14.2%	8.1%	11.4%	14.8%	11.2%	6%	4.7%	8.1%
BMI 6 months previously	25.2±4.9	25.1±4.6	24.6±5.1	25.6±4.7	26±5.3	25.7±5.7	25±4.9	24.7±4.5	24.7±4.5
%BMI ⩾ 30 (every 6 months)	15%	13.2%	15.1%	18.3%	17.6%	17.3%	17.5%	13.1%	12.9%
									
*Malnutrition*	1364								
None	69.1%	81.7%	54.4%	68.8%	65.8%	68%	50.5%	59.8%	73%
Present	30.9%	18.3%	45.6%	31.2%	34.2%	32%	49.5%	40.2%	27%
Moderate	18.6%	11.2%	22.5%	22%	26.3%	16.4%	26.3%	21.9%	18%
Severe	12.2%	7.1%	23.1%	9.2%	7.9%	15.6%	23.2%	18.3%	9%

Abbreviations: BMI=body mass index; PS=performance status; WHO=World Health Organisation; WL=weight loss.

a Leukaemia, lymphoma, myeloma.

b Ovarian and uterine cancers.

c Cancers of the oesophagus, stomach and pancreas; liver carcinomas.

d Prostate, urinary, brain, thyroid, testicular and kidney cancers; trunk and limb sarcomas; melanoma; other thoracic or abdominal tumours; unclassified tumour.

**Table 2B:** Treatments

	**Number of patients**	**Percentage**
*Active treatment*	1246^a^	80.65^a^
Surgery	437 (including 74 combination treatments)	28.3^a^
Chemotherapy	680 (including 173 combination treatments)	44^a^
Radiotherapy	167 (including 112 combination treatments)	10.8^a^
Complication of active treatment	70	4.53
Unknown	77	4.98
		
*Others*	299	19.35
Follow-up examination	116	7.51
Palliative care	147	9.51
End-of-life care	15	0.97
Unknown	21	1.36
		
Total	1545	100.00

a A number of patients received combination regimens with two or more treatments (chemotherapy, radiotherapy and/or surgery). Consequently, the sum of the different treatment groups is superior to the overall number of patients actually receiving active treatment.

**Table 3:** Relationships between malnutrition and clinical data

**Risk factors**	**% Malnutrition**	**Odds ratio**	**95% CI**	***P*-value**
*Gender*				
Female	28	1		
Male	35.3	1.4	1.1–1.8	0.004
				
*Type of hospitalisation*				
Outpatient	20.8	1		
In-patient	32.7	1.8	1.3–2.7	0.001
				
*Age (years)*				
⩽70	29.7			
>70	35			0.08
				
*Type of cancer*				
Breast	18.3	1		
Head and neck	45.6	3.7	2.4–5.8	<0.0001
Colon-rectum	31.2	2	1.3–3.2	0.0019
Haematological	34.2	2.3	1.4–3.8	0.0004
Digestive	49.5	4.4	2.6–7.3	<0.0001
Gynaecological	32	2.1	1.3–3.4	0.0018
Lung	40.2	3	1.8–5.1	<0.0001
				
*Type of stay*				
Curative treatment	26.8	1		
Under evaluation	36.6	1.6	1.0–2.4	0.035
Palliative care	59.2	4	2.7–5.8	<0.0001
				
*Metastases*				
No	27.8	1		
Yes	34.3	1.4	1.1–1.7	0.0093
				
*Radiotherapy*				
No	29.3	1		
Yes	40.1	1.6	1.2–2.2	0.0024
				
*Chemotherapy*				
No	28.9			
Yes	32.4			0.16
				
*Surgery*				
No	31.6			
Yes	26			0.13
				
*Obesity 6 months previously*				
BMI <30	28.5	1		
BMI ⩾30	38.8	1.6	1.2–2.2	0.0032
				
*WHO PS*				
0	15.4	1		
1	24.3	1.8	1.2–2.6	0.0037
2	38	3.4	2.3–4.9	<0.0001
3	50.2	5.5	3.6–8.4	<0.0001
4	46.7	4.8	2.7–8.4	<0.0001
0–1	19.6	1		
2–3–4	43.3	3.1	2.4–4.0	<0.0001
				
**Malnutrition**	**% Antibiotic therapy^a^**			
No	22.8	1		
Yes	35.5	1.9	1.4–2.5	<0.0001

Abbreviations: BMI=body mass index; CI=confidence interval; PS=performance status; WHO=World Health Organisation.

a With the exception of antibiotic prophylaxis for surgery.

**Table 4:** Factors independently associated with malnutrition

**Risk factors**	**Odds ratio**	**95% CI**	***P*-value**
BMI ⩾30	1.58	1.08–2.31	0.018
PS ⩾2	2.71	2.30–6.70	<0.01
Digestive cancer^a^	3.39	1.89–6.10	<0.01
Head and neck cancer	2.28	1.53–3.41	<0.01

Abbreviations: BMI=body mass index; CI=confidence interval; PS=performance status.

a Oesophagus, stomach and pancreas cancers, liver carcinoma.

**Table 5:** Factors independently associated with mortality

**Risk factors**	**Odds ratio**	**95% CI**	***P*-value**
Presence of metastases	2.21	1.3–3.73	0.03
Palliation^a^	3.96	2.17–7.25	<0.001
Evaluation	2.80	1.38–5.69	0.004
Haematological malignancy	2.43	1.17–5.03	0.017
Gynaecological cancer	2.34	1.14–4.83	0.021
Lung cancer	2.85	1.37–5.93	0.005
*WHO*			
PS 2	2.19	1.18–4.05	0.013
PS 3	4.12	2.2–7.72	<0.001
PS 4	8.77	4.08–18.9	<0.001
Severe malnutrition	2.47	1.40–4.36	0.002
Age >70 years	2.01	1.21–3.34	0.007

Abbreviations: CI=confidence interval; PS=performance status; WHO=World Health Organisation.

a All terminally ill patients were dead at 2-months of follow-up.
